# Characterisation of gut microbiota in Malaysian cancer patients using V3-V4 region of 16S rRNA gene sequencing

**DOI:** 10.1038/s41598-025-06983-x

**Published:** 2025-07-01

**Authors:** Siti Farah Norasyikeen Sidi Omar, Yvonne Ai Lian Lim, Ab Rahman Syaza Zafirah, Azdayanti Muslim, Qasim Ayub, Syafinaz Amin-Nordin, Vesudian Narcisse Mary Sither Joseph, Sabri Musa, Timothy Jinam, Romano Ngui

**Affiliations:** 1 Department of Paraclinical Sciences, Faculty of Medicine and Health Sciences, Universiti Malaysia Sarawak, Kota Samarahan, Sarawak, 94300 Malaysia; 2 Department of Parasitology, Faculty of Medicine, Universiti Malaya, Kuala Lumpur, 50603 Malaysia; 3 Department of Paediatrics, Faculty of Medicine, Universiti Malaya, Kuala Lumpur, 50603 Malaysia; 4 Department of Medical Microbiology and Parasitology, Universiti Teknologi MARA (Sungai Buloh Campus), Sungai Buloh, Selangor Darul Ehsan, 47000 Malaysia; 5 Monash University Malaysia Genomics Platform, School of Science, Monash University, Bandar Sunway, Selangor Darul Ehsan, 47500 Malaysia; 6 Department of Medical Microbiology, Faculty of Medicine & Health Sciences, Universiti Putra Malaysia, Serdang, Selangor Darul Ehsan, 43400 Malaysia; 7 Department of Paediatric Dentistry and Orthodontics, Faculty of Dentistry, Universiti Malaya, Kuala Lumpur, 50603 Malaysia

**Keywords:** Gut microbiota, Cancer, 16S rRNA sequencing, Hypervariable V3-V4, Symptomatic and asymptomatic cancer patients, Malaysia, Metagenomics, Microbiome

## Abstract

Recent studies suggested a potential connection between gut microbiota changes and cancer onset. However, conflicting results make it challenging to understand the role of gut microbiota dysbiosis in cancer, particularly in underrepresented populations like those in Southeast Asia. To address this gap, we analysed the diversity and composition of gut microbiota in 65 faecal samples, which included 48 from cancer patients with various malignancies and 17 from healthy controls. Patients were categorised into four groups: symptomatic patients undergoing cancer treatment, asymptomatic pre-treatment and during cancer treatment, and healthy controls. Genomic DNA was extracted, and the V3-V4 region of the 16 S rRNA gene was sequenced. Our findings revealed significant differences in the alpha diversity (*p* ≤ 0.05) between cancer patients and controls. Asymptomatic patients under treatment showed slightly lower alpha diversity than pre-treatment patients, but this difference was not statistically significant (*p* = 0.06). We identified 13 genera with over 20% difference in abundance between patient groups and controls. Asymptomatic patients receiving treatment and pre-treatment patients exhibited enrichment in *Enterococcus*, whereas *Prevotella*,* Faecalibacterium*, *Brevundimonas*, and *Pseudomonas* were significantly reduced compared to controls. Symptomatic patients had higher levels of *Enterococcus* and *Staphylococcus*, while *Ruminococcus* was enriched in asymptomatic patients. These underscore the distinct differences in gut microbiota composition between cancer patients and healthy individuals, particularly in symptomatic cases with potential biomarkers such as *Enterococcus*, *Prevotella*, and *Faecalibacterium*. Our study suggests that cancer treatment may not significantly alter the gut profile of cancer patients. Further research is needed to comprehend the implications of these findings fully.

**Supplementary Information:**

The online version contains supplementary material available at 10.1038/s41598-025-06983-x.

## Introduction

The human gut microbiota is a highly diverse and dynamic community of microorganisms, including bacteria, archaea, fungi, eukaryotic viruses, and bacteriophages, which coexist in a complex ecosystem^1^. The gut microbiota, mainly composed of bacteria, performs essential physiological functions to maintain the body’s stability, such as immunological regulation, nutritional metabolism, and defence against pathogens^2^. Each person’s gut microbiota is unique and is influenced by factors such as diet, medication use, lifestyle, genetics, and environmental conditions^1^. Recent studies have shown a connection between an imbalanced gut microbiota, known as dysbiosis, and the development of various diseases, including cancer^3^.

Cancer is increasingly concerning due to the growing number of new cases^4^. In 2022, cancer caused 9.7 million deaths globally^4^. Recent advancements in high-throughput sequencing technologies have allowed researchers to study the link between gut microbiota and various diseases, including cancer. Studies have found that cancer patients have a different gut microbiota composition than healthy individuals^5,6^. It has been observed that cancer patients often have lower microbial diversity, which is associated with adverse outcomes such as increased inflammation^7^, weakened immune responses, and impaired metabolism. These factors can contribute to cancer progression and poor treatment outcomes^8,9^. Additionally, cancer treatment methods such as immunotherapy, chemotherapy, and targeted therapy can potentially influence the composition and diversity of gut microbes^10^.

The gut microbiota has been linked to different types of cancer. In colorectal cancer, patients often show specific microbial patterns. These patterns include an overrepresentation of pro-inflammatory and potentially carcinogenic bacteria such as *Fusobacterium nucleatum*, *Escherichia coli*, and *Bacteroides fragilis*, along with a decrease in beneficial microbes like *Bifidobacterium* and *Lactobacillus*^11^. Recent studies have shown that the gut microbiota can also be affected by diseases beyond the intestines. Several studies have shown inconsistent findings on the mechanisms underlying gut microbiota dysbiosis in cancer^12–14^. Therefore, studying the gut microbiota of cancer patients is highly relevant, as it plays a critical role in understanding cancer development, progression, response to treatment, and potential for novel therapeutic strategies. However, there is a scarcity of studies focusing on Malaysian cancer patients^15–17^, with the existing research predominantly featuring Western and East Asian populations, limiting the generalizability of findings to other ethnic and geographical groups. Given Malaysia’s rising cancer incidence^18^, it is crucial to investigate the role of gut microbiota in cancer within this specific demographic. Our study aims to examine the diversity and composition of gut microbiota in Malaysian cancer patients and compare it with healthy controls. We will present our findings on the gut microbiota profiles of Malaysian cancer patients and explore potential microbial biomarkers and dysbiosis associated with cancer. We hope to gain a deeper understanding of the complex interactions between gut microbiota and cancer within the Malaysian context using advanced metagenomic sequencing techniques. This could lead to personalised microbiota-based interventions and the identification of new therapeutic strategies.

## Materials and methods

### Ethical approval and consent to participate

The research obtained approval from the Medical Ethics Committee of the Universiti Malaya Medical Centre (UMMC) (MREC ID No. 2019528-7454). All methods complied with the guidelines and regulations approved by the UMMC Medical Ethics Committee. Before the research began, participants received detailed information about the study’s purpose and procedures. They were assured that there were no inherent risks associated with the methods used and were promised confidentiality and anonymity throughout the study. Participation was voluntary, and participants had the right to withdraw from the research without facing any consequences. Informed consent was obtained through written signatures or, in the case of illiterate participants, verbal affirmation followed by a thumbprint. For participants under 16, informed consent was obtained from parents or legal guardians using the same methods.

### Study populations

Generally, the sample collection and processing details have been presented elsewhere^19^. Briefly, 53 cancer patients with various malignancies were recruited from the Oncology Unit at the Universiti Malaya Medical Centre (UMMC) using convenient sampling. To be eligible, patients had to be at least 1 year old, have a confirmed cancer diagnosis, and provide written consent. Patients were either newly diagnosed or undergoing active treatment at the time of recruitment and had not taken antibiotics within one month before sample collection. Those who did not meet these criteria were excluded from the study. Patients were either newly diagnosed or undergoing active treatment at the time of recruitment. Those who presented comorbidities such as Type 2 Diabetes, obesity, or cardiovascular disease and had taken antibiotics within one month before sample collection was excluded from the study.

The patients were diagnosed with ten different types of cancers, which were classified based on Disease Ontology and the primary site of tumours. Fourteen patients experienced gastrointestinal (GI) symptoms, defined as one or more symptoms such as diarrhoea, nausea, vomiting, and abdominal cramps. These patients were adjusted for downstream analysis to remove bias by grouping them as symptomatic or asymptomatic. Demographic data, including age, gender, personal identification, diagnosis, and date of cancer therapy, were obtained from the medical records available to the investigator. Additionally, 17 healthy Malaysian individuals were included as healthy controls. These individuals served as a baseline for healthy gut microbiota comparison. They were assumed to have similar dietary and lifestyle habits, partially controlling for environmental effects on the gut microbiota.

### Sample collection, DNA extraction and 16 S ribosomal RNA (rRNA) gene sequencing

Fresh faecal samples were collected in a sterile screw-capped container and immediately stored at −80 °C before DNA extraction. Genomic DNA was extracted from the faecal sample by bead-beating extraction using FavorPrep™ Stool DNA Isolation Mini kit (Favorgen^®^, Taiwan) following the manufacturer’s protocol. The extracted DNA concentration and quality were measured using spectrophotometry (NanoDrop Technologies, USA) and stored at −20 °C until further processing.

The V3-V4 region of the 16S ribosomal RNA (rRNA) gene from extracted genomic DNA was PCR amplified using a similar pipeline to that used in Siti Farah Norasyikeen et al.^19^. Briefly, the forward primers 341 F (5’-TCG TCG GCA GCG TCA GAT GTG TAT AAG AGA CAC CTA CGG GNG GCW GCA G-3’) and reverse primers 785R (5’-GTC TCG TGG GCT CGG AGA TGT GTA TAA GAG ACA GGA CTA CHV GGG TAT CTA ATC C-3’) containing Illumina adapter overhang nucleotide sequences (forward overhang: (5’ TCG TCG GCA GCG TCA GAT GTG TAT AAG AGA CAG-[locus specific sequence]) and reverse overhang: (5’ GTC TCG TGG GCT CGG AGA TGT GTA TAA GAG ACA G-[locus specific sequence])) were used to amplify 460 bp fragments of V3-V4 regions of the bacterial 16 S rRNA gene^20^.

The samples library was constructed using KAPA HiFi HotStart, and Illumina Miseq sequencing was performed at Monash University Malaysia Genomics Platform. The barcoded PCR amplicon was pooled in equimolar ratio for the 2 × 250 bp paired-end sequences with an insert size of 450 bp and purified with AMP XP beads (Beckman Coulter, US). The extracted DNA quantity and quality were analysed by a fluorometer with dsDNA binding dyes using the Agilent DNA 1000 Kit (Agilent, Germany).

### Metagenomic sequence processing and quality control

The sequence reads were pre-processed as described previously^19^. Briefly, raw FASTQ files containing the paired-end sequence were imported as manifest files into Quantitative Insights into Microbial Ecology 2 (QIIME2) software package version 2024.5 for demultiplexing, sequence processing, quality filtering and analysis. Following data import, the raw sequence data were demultiplexed to remove the barcode sequence. The demultiplexed paired ends were assembled, and paired-end reads with overlaps of 10 bp were trimmed for Illumina adapters and the primers. Reads that could not be assembled and contained 2 nucleotide mismatches in primer matching were discarded. The trimmed paired-end read was joined and filtered based on the quality score. Joined reads were truncated at any site, receiving an average quality score of < 20, and the truncated reads shorter than 50 bp containing ambiguous characters were removed. The joined reads were denoised using Deblur (q2-deblur plugin)^21^ to filter out noisy sequences, remove chimeric sequences, remove singletons and dereplicate the sequences to produce feature data and a table known as Amplicon Sequence Variant (ASV). ASVs that appear with less than 0.25% abundances were also removed to mitigate inflation of microbial richness due to sequencing errors. Total Sum Scaling (TSS) was performed on the ASVs feature data, which was normalised using Total Sum Scaling (TSS) to ensure even sampling across all samples for downstream diversity analysis. A total of 70 paired-end sequence reads were processed for metagenomic sequencing, resulting in 3,241 474 quality-filtered sequences with an average of 46,306 sequences per sample obtained after quality control and contamination removal (Table S1).

### Microbial community diversity analysis and taxonomic profiling

Alpha diversity, which measures species richness and evenness within individual samples, was assessed using observed species richness, the Shannon index and Shannon Effective Number, calculated from normalised ASVs. Beta diversity, a measure of variability in community composition, was measured using Bray-Curtis dissimilarity to measure microbial dissimilarity in the samples, and principal coordinate analysis (PCoA) was computed based on the distance metrics generated^22,23^. The ASVs were taxonomically classified against the pre-trained SILVA release 138.2 database with 99% OTUs reference sequences trimmed for 16 S rRNA V3-V4 regions using RESCRIPt. Taxonomic analyses were conducted at both phyla and genus levels.

### Differential abundance of microbial communities

To identify key bacterial taxa in cancer patients, differentially abundant bacteria were analysed pairwise using the Linear Discriminant Analysis Effect Size (LEfSe) method via the online interface Galaxy (http://huttenhower.sph.harvard.edu/galaxy/). The analysis compared cancer patients, adjusted for GI symptoms, against a baseline control group. Patients exhibiting GI symptoms were excluded to minimise environmental effects on the results. Initially, LEfSe identified statistically significant features among the biological classes. This was followed by a non-parametric factorial Kruskal-Wallis rank-sum test to detect differences in bacterial taxa. Subsequently, a linear discriminant analysis model was applied to estimate the effect sizes of these features, determining whether they conformed to the expected behaviour of the different biological classes.

### Statistical analysis

Demographic data, including personal identification, age, gender, symptoms, diagnosis, cancer treatment, and date of cancer therapy, were keyed into the Statistical Package for the Social Sciences software (SPSS version 26.0, SPSS Inc., Chicago, IL, USA). These demographic data were treated as categorical variables and presented as frequency (per cent). When appropriate, a Chi-square or Fisher’s exact test was conducted to identify any differences among the variables. A p-value of less than 0.05 was considered statistically significant.

Alpha diversity group significance was calculated using the Kruskal-Wallis pairwise statistic in the QIIME2 plugin. Bray-Curtis dissimilarity for beta diversity was calculated pairwise between sample groups using permutational multivariate analysis of variance (PERMANOVA) with 999 permutations using QIIME2.

Statistical significance of the differentially abundant genera between three or more groups was performed using the Kruskal-Wallis test in R. For pairwise comparisons between groups, the Wilcoxon rank-sum test was performed following Kruskal-Wallis to measure significance in abundance. The false discovery rates of the resulting p-values were corrected using the p.adjust function with the Benjamin-Hochberg method implemented in R. A *p* < 0.05 was considered significant. All results were visualised using the “ggplot2” package implemented in R.

## Results

### Study population characteristics

**Fig. 1:**
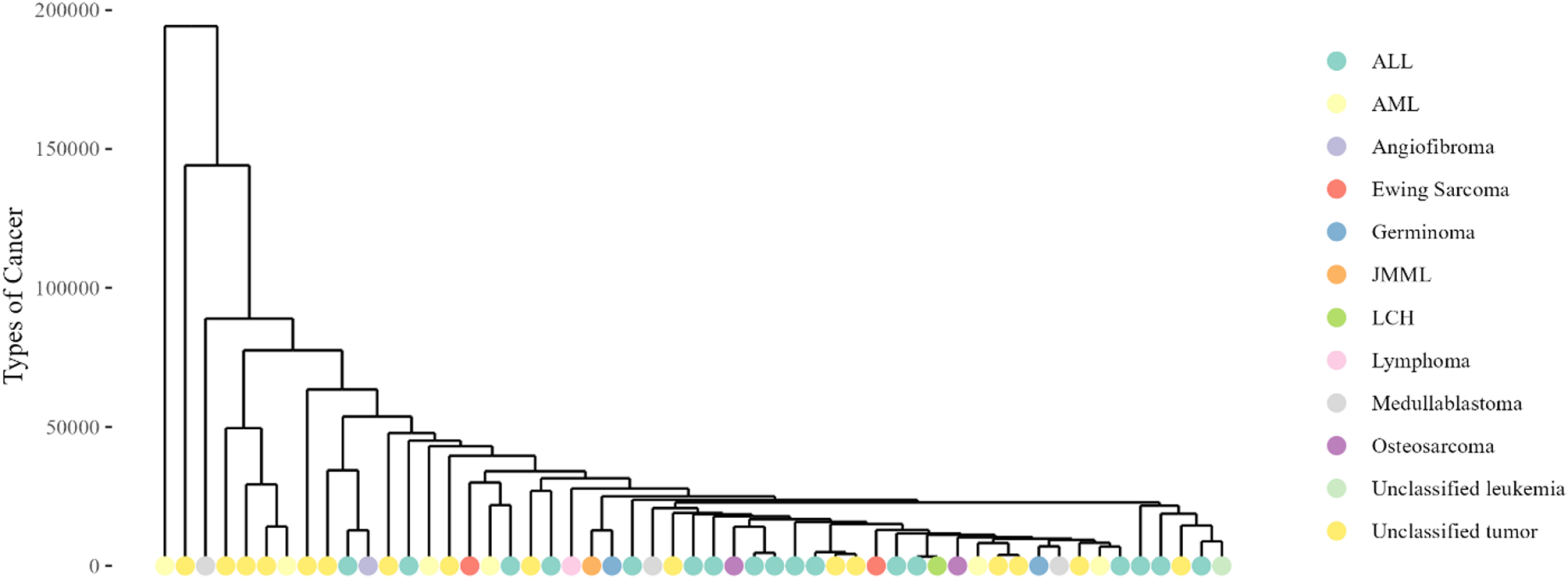
Dendrogram shows sample clustering based on taxonomic profiles with slight clustering of samples measured based on the types of cancer. ALL = acute lymphoblastic leukaemia; AML = acute myeloid leukaemia; JMML = juvenile myelomonocytic leukaemia; LCH = Langerhans cell histiocytosis.

Fifty-three cancer patients and 17 healthy controls were included in this study. Of these, the 36 cancer patients were classified based on their primary site of tumours: medulloblastoma (*n* = 3), Ewing sarcoma (*n* = 2), osteosarcoma (*n* = 2), germinoma (*n* = 2), angiofibroma (*n* = 1), langerhans cell histiocytosis (LCH) (*n* = 1), acute lymphoblastic leukaemia (ALL) (*n* = 17), acute myeloid leukaemia (*n* = 6), lymphoma (*n* = 1) and juvenile myelomonocytic leukaemia (JMML) (*n* = 1). The remaining 17 cancer patients were classified as unclassified solid tumours (*n* = 16) and leukaemia (*n* = 1) due to the inability to retrieve their data from the medical records. The distribution of samples across cancer types was significantly different (*p* = 0.047) (Table 1). Based on dendrogram clustering on their taxonomic profiles, intra-patient samples with similar cancer types do not necessarily cluster together, demonstrating inter-individual variability in the study groups (Fig. 1).

**Table 1:** Baseline characteristics of cancer patients.

Characteristics	Overall (*N* = 53)	Pre-treatment (*N* = 13)	During treatment (*N* = 40)	*p*-value
**Age** **Range** **Median (IQR)** **Mean (SD)**	1–96 13 (4–47.5) 24.92 (26.394)	2–17 6 (3.5–13) 8 (5.4)	1–96 16 (4.3–55.5) 30.4 (28.17)	^a^0.703
**Age groups** **≤ 3** **4–6** **7–12** **13–18** **19–39** **40–59** **≥ 60**	10 (18.9%) 9 (17.0%) 7 (13.2%) 8 (15.1%) 4 (7.5%) 7 (13.2%) 8 (15.1%)	3 (23.1%) 4 (30.8%) 3 (23.1%) 3 (23.1%) 0 (0.0%) 0 (0.0%) 0 (0.0%)	7 (17.5%) 5 (12,5%) 4 (10.0%) 5 (12.5%) 4 (10.0%) 7 (17.5%) 8 (20.0%)	^b^0.114
**Gender** **Male** **Female**	32 (60.4%) 21 (39.6%)	7 (53.8%) 6 (46.2%)	25 (62.5%) 15 (37.5%)	^b^0.579
**Gastrointestinal symptoms** **Asymptomatic** **Symptomatic**	34 (64.2%) 19 (35.8%)	13 (100.0%) 0 (0.0%)	21 (52.5%) 19 (47.5%)	^b,^*0.002
**Cancer Type** **Solid tumour** **Uncharacterised solid tumour** **Medulloblastoma** **Ewing Sarcoma** **Osteosarcoma** **Germinoma** **Angiofibroma** **LCH** **Haematological malignancies** **ALL** **AML** **Lymphoma** **JMML** **Uncharacterised leukaemia**	16 (30.2%) 3 (5.7%) 2 (3.8%) 2 (3.8%) 2 (3.8%) 1 (1.9%) 1 (1.9%) 17 (32.1%) 6 (11.3%) 1 (1.9%) 1 (1.9%) 1 (1.9%)	1 (7.7%) 2 (15.4%) 1 (7.7%) 1 (7.7%) 0 (0.0%) 1 (7.7%) 1 (7.7%) 5 (38.5%) 0 (0.0%) 0 (0.0%) 0 (0.0%) 1 (7.7%)	15 (37.5%) 1 (2.5%) 1 (2.5%) 1 (2.5%) 2 (5.0%) 0 (0.0%) 0 (0.0%) 12 (30.0%) 6 (15.0%) 1 (2.5%) 1 (2.5%) 0 (0.0%)	^b*^0.047
**Cancer Group** **Solid tumour** **Haematological malignancies**	28 (52.8%) 25 (47.2%)	7 (53.8%) 6 (46.2%)	21 (52.5%) 19 (47.5%)	^b^0.933

N = Total number of samples; n = number of samples examined; IQR = Interquartile range; SD = Standard deviation; ALL = acute lymphoblastic leukaemia; AML = acute myeloid leukaemia; JMML = juvenile myelomonocytic leukaemia; LCH = Langerhans cell histiocytosis. *The distribution of pre-treatment and during-treatment cancer patients based on their gastrointestinal symptoms was significantly different based on the Chi-square test (*p* < 0.05). ^a^The distribution of age across treatment groups was measured with the Kruskal-Wallis test (*p* > 0.05). ^b^The distribution of age groups, gender, GI symptoms, cancer type and cancer groups across treatment groups was measured with a Chi-square test (*p* > 0.05).

Most of the samples (*n* = 40) were from patients treated with immunotherapy or chemotherapy, while 13 cancer patients were newly diagnosed and had not yet received medical intervention. There were no significant differences in age groups (*p* = 0.114), gender (*p* = 0.579), or cancer group (*p* = 0.933) between the two groups. Furthermore, cancer patients receiving treatment were either symptomatic (presenting with, for example, diarrhoea or more than one gastrointestinal symptom) (*n* = 19) or asymptomatic (*n* = 21), with a significant difference (*p* = 0.002) in distribution compared to the pre-treatment group, which mainly consisted of asymptomatic patients (*n* = 13) (Table 2).

**Table 2:** Pairwise PERMANOVA statistics based on Bray-Curtis distances.

Pairwise		PERMANOVA		
Group 1	Group 2	pseudo-F	*p*-value	q-value
**Control**	**Asymptomatic Pre-treatment**	2.456	0.001*	0.002*
	**Asymptomatic During treatment**	2.842	0.001*	0.002*
	**Symptomatic treatment**	4.203	0.001*	0.002*
**Asymptomatic Pre-treatment**	**Asymptomatic During treatment**	0.997	0.48	0.48
	**Symptomatic During treatment**	2.446	0.001*	0.002*
**Asymptomatic During treatment**	**Symptomatic During treatment**	1.82	0.004*	0.005*

### Distinct gut microbiota diversity between cancer patients and healthy controls

We examined the gut microbial diversity patterns between cancer patients receiving treatment and those not yet receiving treatment. Previous research has shown that gastrointestinal (GI) symptoms, especially diarrhoea, can significantly impact the gut microbiota^24,25^. To minimise the effects of environmental factors on gut diversity, we accounted for GI symptoms when grouping the samples. As a result, we classified the samples into four groups: symptomatic and receiving treatment (*n* = 14), asymptomatic and receiving treatment (*n* = 21), asymptomatic and pre-treatment (*n* = 13), and control (*n* = 17).

**Fig. 2:**
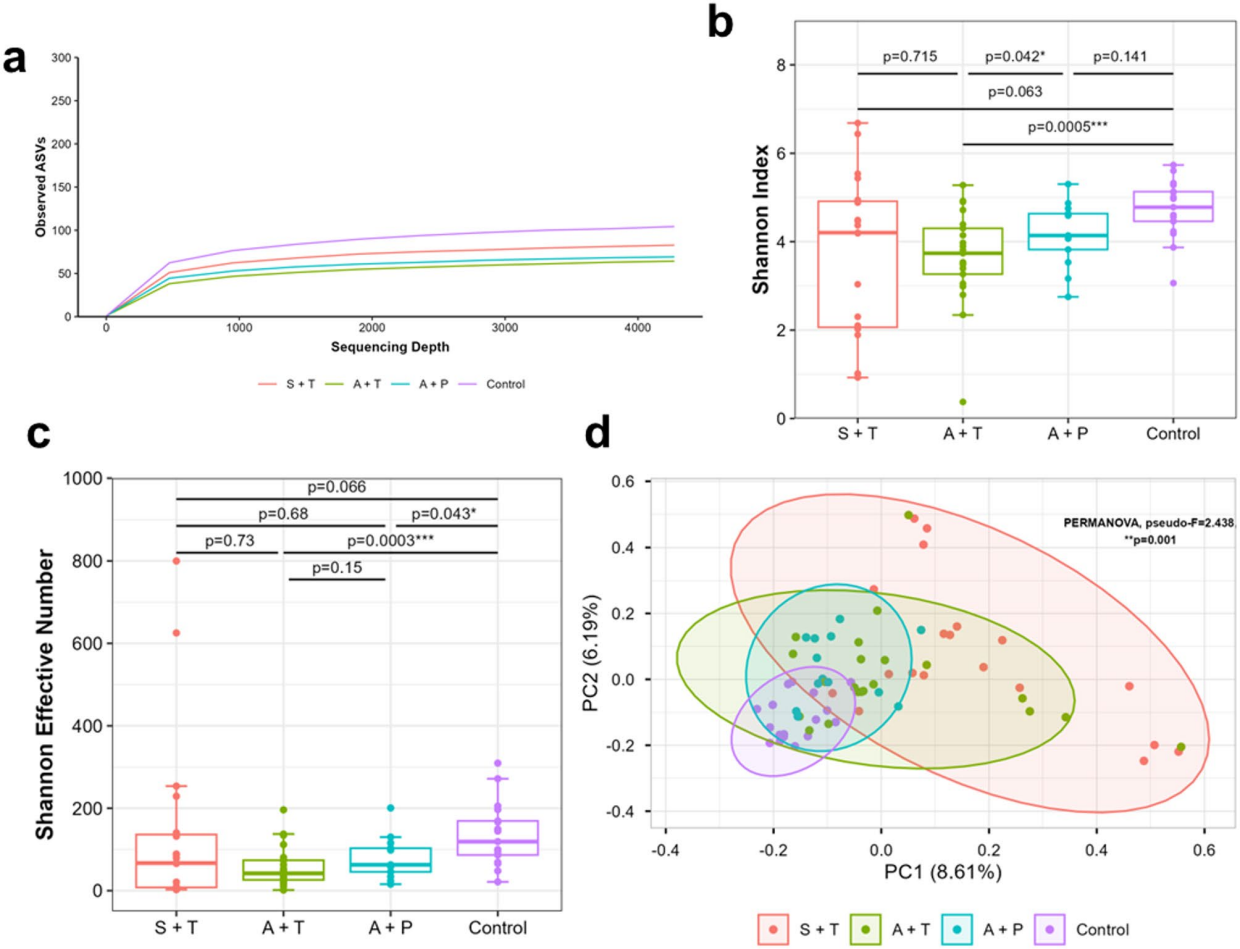
Alpha and beta diversity measured on cancer patients at pre-treatment (P) and treatment (T) adjusted for GI symptoms against healthy control. **(a)** Observed species richness at minimum sample depth of 4273, **(b)** Shannon effective index and **(c)** Shannon Effective Number. **(d)** PCoA plot based on the Bray-Curtis dissimilarity showed distinct clustering between cancer patients and controls. *Boxplot depicts the median, interquartile and extremities of the values. p-value calculated using Kruskal-Wallis t-test with *p ≤ 0.05, **p ≤ 0.01, and ***p ≤ 0.001.

Alpha diversity analysis was conducted using observed species richness, the Shannon index and Shannon Effective Number (Fig. 2a, b, c). The control group have higher microbial richness, followed by symptomatic cancer patients receiving treatment, asymptomatic cancer patients that are not receiving treatment and asymptomatic cancer patients receiving treatment (Fig. 2a). The results revealed a significant reduction in microbial diversity richness in symptomatic cancer patients receiving treatment (Shannon index: *p* = 0.127; Shannon Effective Number: *p* = 0.066)), asymptomatic cancer patients receiving treatment (Shannon index: *p* = 0.003; Shannon Effective Number: *p* = 0.0003), and pre-treatment (Shannon index: *p* = 0.127; Shannon Effective Number: *p* = 0.043) when compared to the control group. Asymptomatic patients receiving treatment had a significantly lower median alpha diversity value than pre-treatment patients (Shannon index: *p* = 0.042; Shannon Effective Number: *p* = 0.15). Interestingly, higher median alpha diversity was observed in symptomatic patients compared to asymptomatic patients receiving treatment, but was not statistically significant (Shannon index: *p* = 0.715; Shannon Effective Number: *p* = 0.73) (Fig. 2b, c).

We used multivariate statistical analysis to compare the differences in microbial community structures (Beta diversity) using Bray-Curtis dissimilarity distances. We found significant variation in the microbial composition among the four groups, as shown by the PERMANOVA test (pseudo-F = 2.438, *p* = 0.001) (Fig. 2d). The Principal Coordinate Analysis (PCoA) plot also demonstrated that the three patient groups (symptomatic and receiving treatment, asymptomatic and receiving treatment, and asymptomatic and pre-treatment) significantly differed from the control, indicating differences in microbiota composition (Fig. 2d). Symptomatic cancer patients receiving treatment showed the most difference from the control group (PERMANOVA: pseudo-F = 4.203, *p* = 0.002), followed by asymptomatic patients receiving treatment (PERMANOVA: pseudo-F = 2.842, *p* = 0.002) and pre-treatment patients (PERMANOVA: pseudo-F = 2.456, *p* = 0.002). Although there was some difference and spread between asymptomatic cancer patients receiving treatment and pre-treatment, the results were not statistically significant (PERMANOVA: pseudo-F = 0.997, *p* = 0.48; ANOSIM: *R* = 0.078, *p* = 0.946) (Table 2).

### Identification of specific taxa related to cancer treatment response

**Fig. 3:**
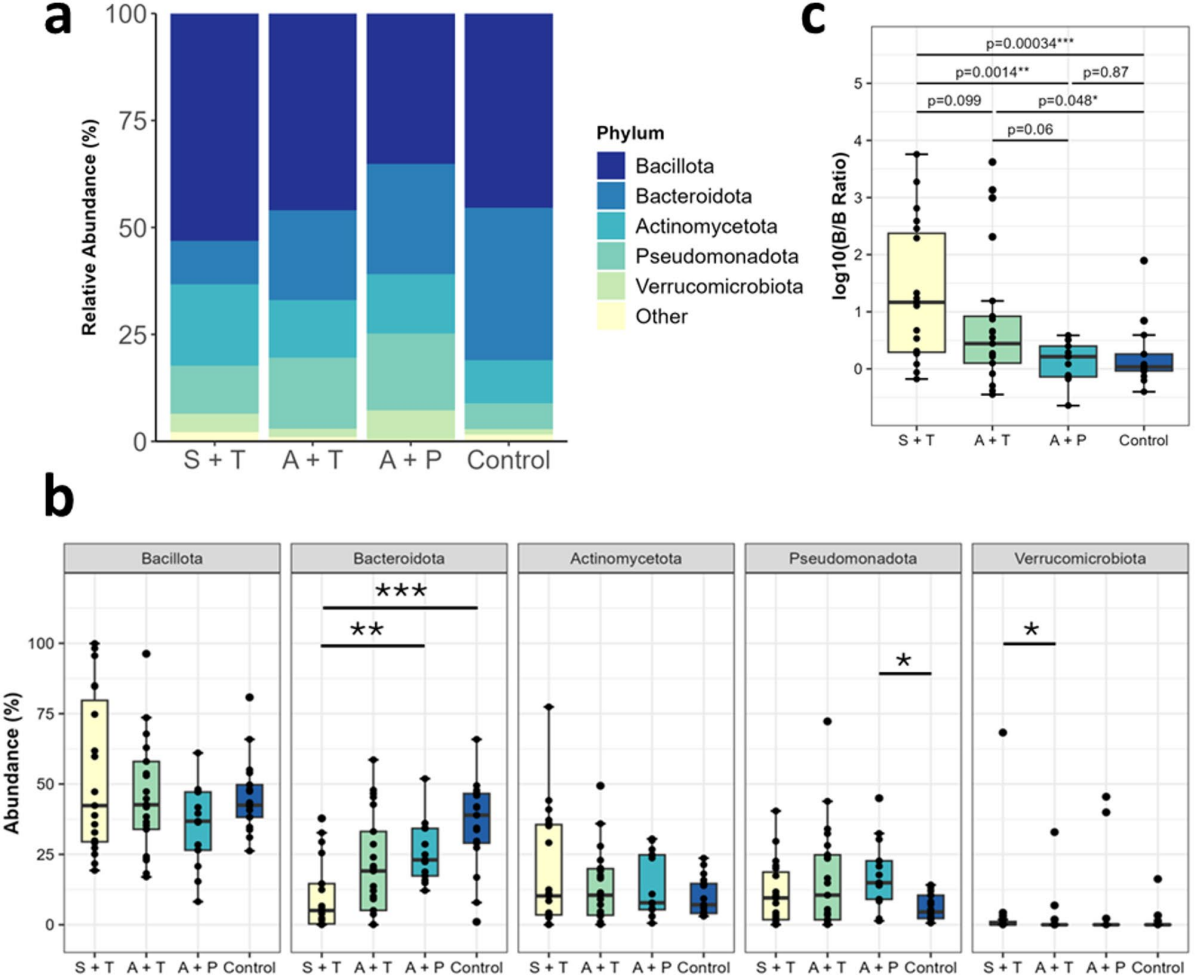
Taxonomic analysis of gut microbiota of cancer patients. **(a)** Taxonomic bar plot showing comparison of microbial composition at phylum level in symptomatic patients who are receiving treatment (S + T), asymptomatic and receiving treatment (A + T), asymptomatic and pre-treatment (A + P) and control. **(b)**. Top 5 phyla with altered relative abundance in cancer patients at pre-treatment (P) and treatment (T) adjusted by GI symptoms. Pairwise comparison between two groups was marked as asterisk for significant differences in relative abundance based on Wilcoxon rank sum test (*p ≤ 0.05, ** *p* ≤ 0.01, *** *p* ≤ 0.001). **(c)** *Bacillota*/*Bacteroidota* (B/B) ratio in abundance of cancer patients and control.

When looking at the phylum level, the most abundant phylum across all 70 samples was *Bacillota* (53.1%), followed by *Bacteroidota* (35.6%), *Actinomycetota* (19.0%), *Psedomonadota* (18.0%), and *Verrucomicrobiota* (6.75%) (Fig. 3a). Symptomatic patients receiving treatment had the highest average relative abundance of *Bacillota* (53.1%), followed by asymptomatic patients receiving treatment (46.0%), the control group (45.4%), and pre-treatment patients (35.2%). Conversely, *Bacteroidota* decreased, and its highest abundance was seen in the control group (35.6%), followed by asymptomatic pre-treatment patients (25.7%) and those receiving treatment (21.0%), with symptomatic patients receiving treatment having the lowest abundance (10.2%) (Fig. 3b). Additionally, it was observed that bacteria from the *Pseudomonadota* phylum were significantly more abundant in asymptomatic pre-treatment patients (18%) compared to the control group (6.1%) (Wilcoxon rank sum test, *p* = 0.008).

**Fig. 4:**
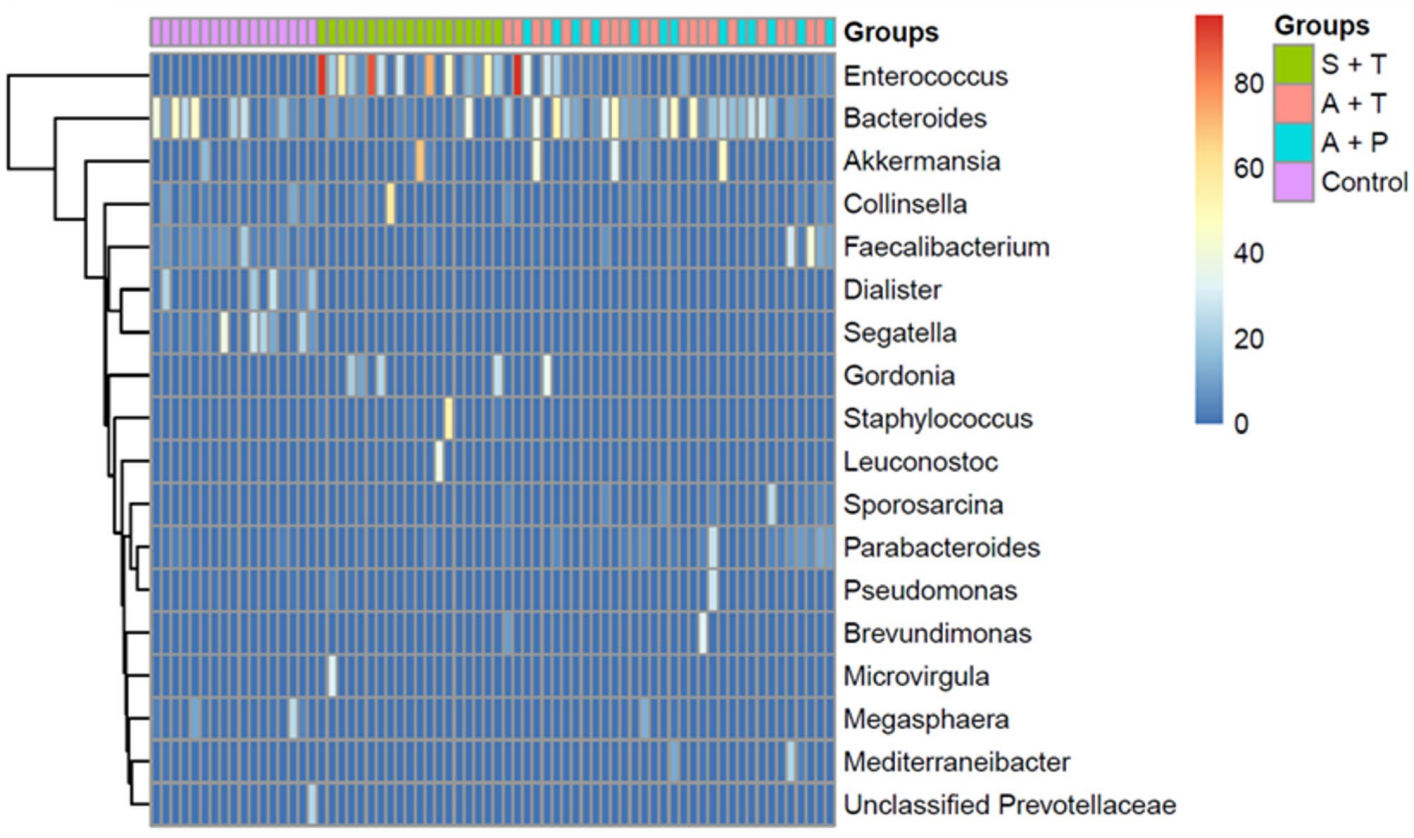
Heatmap of differentially abundant genus between S + T, A + T, A + P and control with more than 20% abundance (Kruskal-Wallis test, *p* ≤ 0.05).

When comparing the *Bacillota*/*Bacteroidota* (B/B) ratios, it was found that symptomatic patients receiving treatment had a significantly higher ratio than asymptomatic patients receiving treatment (*p* = 0.099) and pre-treatment patients (*p* = 0.0014), as well as the control group (*p* ≤ 0.001). However, no differences in the B/B ratios value were observed in asymptomatic patients receiving treatment and pre-treatment compared to the control group (Fig. 3c). At the genus level, we found 18 differentially abundant species based on taxa that were 20% more abundant in the overall taxa, as determined by the Kruskal-Wallis t-test (*p* ≤ 0.05) (Fig. 4).

**Fig. 5:**
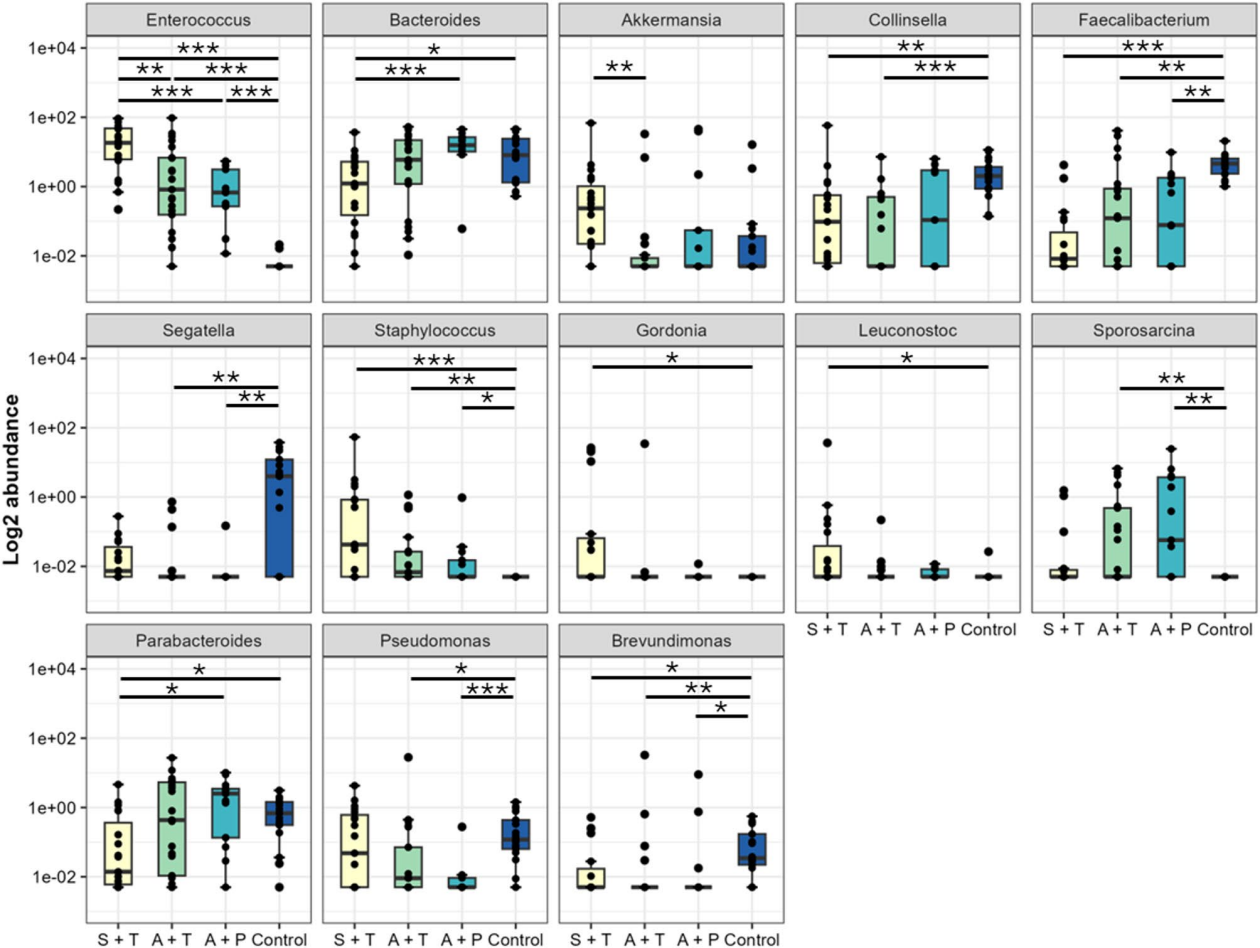
Genera with significant abundance between cancer patients at pre-treatment (P) and treatment (T) adjusted by GI symptoms and control. Genera with altered relative abundance in cancer patients by pairwise comparison between two groups was marked as asterisk for significant differences based on Wilcoxon rank sum test (*p ≤ 0.05, ** *p* ≤ 0.01, *** *p* ≤ 0.001).

Asymptomatic pre-treatment patients had higher levels of bacteria from the *Enterococcus*,* Staphylococcus* and *Sporosarcina* genus. In contrast, levels of *Faecalibacterium*,* Segatella*,* Pseudomonas*, and *Brevundimonas* were significantly reduced compared to the control group (Fig. 5). Asymptomatic patients undergoing treatment showed enrichment in *Enterococcus* and *Staphylococcus*, and reduced levels of *Collinsella*, *Faecalibacterium*,* Segatella*,* Sporosarcina*,* Psedomonas* and *Brevundimonas*, compared to the control group. We compare patients receiving and pre-treatment to determine differences in microbial populations in cancer treatment. We observed reduced bacteria from the genera *Bacteroides*,* Collinsella*,* Sporosarcina* and *Parabacteroides*, but they were not differentially abundant between the asymptomatic groups. Meanwhile, symptomatic patients undergoing treatment observed significantly higher *Enterococcus* and *Staphyloccocus* with a reduction in *Bacteroides*, *Collinsella*, *Faecalibacterium*, *Gordonia*, *Leuconostoc*,* Parabacteroides* and *Brevundimonas* compared to control. *Akkermansia* was also increased in symptomatic patients, but this was not statistically significant. To determine differences in taxa between symptomatic and asymptomatic patients, we compared the taxa in patients undergoing treatment. We observed that *Enterococcus* and *Akkermansia* were more significantly abundant in symptomatic patients. Meanwhile, *Bacteroides*, *Faecalibacterium* and Parabacteroides were more abundant in asymptomatic patients, but were not statistically significant.

### Differential microbial profiles in cancer patients compared to healthy controls

**Fig. 6:**
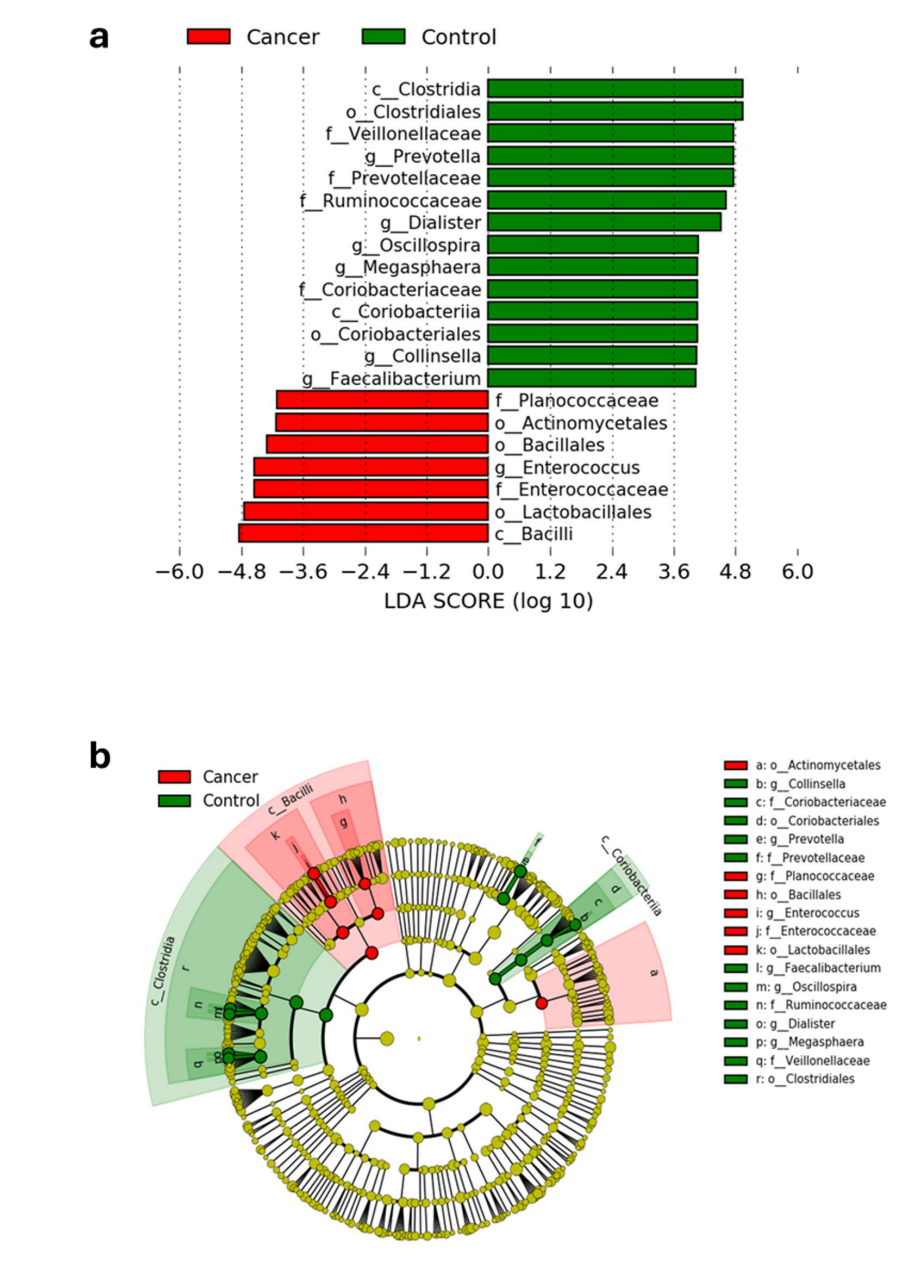
Different abundances of microbial communities between cancer patients and healthy control. **(a)** Linear discriminative analysis (LDA) effect size (LEfSe) analysis between asymptomatic cancer patients (red) and healthy controls (green). **(b)** A cladogram displays the connection between the significantly different taxa at different levels with the clade as a branch of organisms that share a common ancestor.

We utilised the LEfSe (linear discriminant analysis effect size) method to pinpoint significant biomarkers. Our study compared 53 cancer patients undergoing treatment or pre-treatment with 17 healthy individuals. We grouped the two sets of cancer patients since beta diversity analysis revealed no distinctions between them. We excluded cancer patients displaying symptoms to eliminate any environmental factors that could affect gut bacteria. The analysis showed that certain types of bacteria were more common in cancer patients with an LDA score greater than 4.0, including *Actinomycetales*,* Planoconcave*, and *Enterococcus*. On the other hand, the control group had higher levels of *Prevotella*,* Dialister*,* Oscillospira*,* Megasphaera*,* Collinsella*, and *Faecalibacterium* (Fig. 6).

## Discussion

Numerous studies have reported significant changes in the gut microbiota in cancer patients, but most do not focus on the Malaysian populations^13,14,26–30^. There is limited research on the gut microbiota of Malaysian cancer patients. This study aimed to assess the gut microbiota profiles of cancer patients in Malaysia, particularly in the urban population of Kuala Lumpur. The cohort included patients undergoing cancer treatment as well as newly diagnosed patients with various cancer types, as it was challenging to recruit patients with specific types of cancer. Samples from patients with diarrhoea or multiple gastrointestinal symptoms were also included to evaluate differences in taxa in cancer patients experiencing dysbiosis. High-throughput sequencing targeting the V3 and V4 regions of bacterial genomic DNA from faecal samples revealed significant differences in gut microbiota diversity and composition between cancer patients and healthy controls.

The study found that patients receiving treatment and those without symptoms before treatment had significantly reduced microbial diversity and substantial variability in microbial composition. These results support the hypothesis that gut microbiota diversity decreases in disease states, leading to distinct microbial compositions compared to healthy individuals^31^. Despite previous reports indicating that cancer treatment influences gut microbiota profiles^32,33^, this study did not observe significant differences in microbial species richness and composition between patients receiving treatment and those without symptoms before treatment. This lack of significant differences may be influenced by various factors, including cancer stage, number of chemotherapy cycles, types of treatment, and tumour locations. Additionally, since samples were not collected during the intensive chemotherapy phase, the gut microbiota may have partly recovered, as suggested by previous studies^28^. However, these observations cannot be confirmed without samples collected during this phase.

Some studies have reported similar results to ours^16,26,30,34^. According to these studies, gut microbiota dysbiosis can occur before the initiation of chemotherapy^26,27^ and persist throughout the first year of chemotherapy^28,35^. Hence, our pre-treatment groups may already be experiencing dysbiosis, as indicated by the low median alpha diversity compared to healthy controls. The observed changes in pre-treatment patients could result from the disease’s immunomodulation, although the mechanisms underlying immune derangement remain unclear and require further investigation.

While we did not observe significant differences in diversity and microbiota composition between asymptomatic and symptomatic cancer patients, we observed that cancer patients undergoing treatment, particularly symptomatic individuals, exhibited higher Shannon diversity and greater variability in microbial composition. Diarrhoea, a common symptom among these patients, likely contributed to these shifts by increasing bowel movements, destabilising the gut environment and increasing stool water content, which can contribute to the higher alpha diversity observed^24,25^. Additionally, the high alpha diversity in symptomatic patients may reflect a dysbiotic state characterised by pathobiont overgrowth (such as *Enterococcus* and *Staphylococcus*) with elevated *Akkermansia*, a mucin-degrading bacterium linked to gut barrier repair. This paradoxical coexistence of inflammation-associated taxa and putative beneficial microbes could indicate simultaneous treatment-induced gut inflammation and early recovery processes.

In our study, *Bacillota* and *Bacteroidota* were the most predominant phyla. This is consistent with findings from other studies on human gut microbiota^26,29^. We observed no significant differences in the *Bacillota*/*Bacteroidota* (B/B) ratios between asymptomatic pre-treatment patients and healthy controls. However, cancer patients undergoing treatment, especially those with symptoms, showed an increased abundance of *Bacillota* and a decreased abundance of *Bacteroidota*. This shift in microbial composition aligns with previous studies where children with acute lymphoblastic leukaemia (ALL) had gut microbiota dominated by *Bacteroidota* before chemotherapy, which decreased post-treatment^15,28^. The B/B ratio is often used to indicate microbial imbalances and dysbiosis^36,37,38^ and is frequently employed to reflect gut microbiota disorders^31^. The elevated B/B ratio observed in our study has also been linked to other conditions, such as irritable bowel syndrome (IBS)^39,40^. DeFrees and Bailey^41^ speculated that alterations in the bacterial phyla of IBS patients might be related to changes in epithelial permeability and gut inflammation. Additionally, our findings indicated a significant enrichment of *Pseudomonadota*, particularly in asymptomatic patients. Hakim et al.^28^ reported that increased *Pseudomonadota*are associated with a heightened risk of developing neutropenia. The role of *Pseudomonadota* as markers of gut microbiota imbalance and increased disease risk has been well-documented in both immunocompetent and immunocompromised patients^42–44^.

Further analysis at the genus level revealed that bacteria from the genus *Enterococcus* were more prevalent in cancer patients compared to controls, especially in symptomatic patients. Previous studies have indicated that members of this genus increase following chemotherapy^45^. However, our study did not find significant differences in the abundance of *Enterococcus* between patients receiving treatment and those not yet receiving treatment. This lack of difference may be influenced by various factors previously discussed, which we acknowledge as limitations of our study. Nonetheless, we observed significant increases in the abundance of *Enterococcus* and *Staphylococcus* in symptomatic patients. Hakim et al.^28^ confirmed that increasing these taxa may predispose patients to a greater risk of developing neutropenia and diarrheal illness.

*Enterococcus* are lactic acid-producing bacteria commonly found in the gastrointestinal tract of healthy individuals^46^. There is increasing evidence regarding the role of *Enterococcus* in cancer treatment. Some species, such as *Enterococcus faecalis* and *Enterococcus faecium*, can produce enterocin, which has antimicrobial and anticancer properties and promotes the activity of lymphocytes responsible for recognising and destroying cancer cells^47–50^. On the other hand, *Enterococcus* (particularly *E. faecalis* and *E. faecium*) can also cause opportunistic nosocomial infections^46^. *E. faecalis* accounts for 80–90% of human clinical enterococcal infections, while *E. faecium* causes 5–15%^51^. In colorectal cancer, *E. faecalis* has been shown to produce metabolites that compromise the intestinal barrier and induce inflammation^52^. According to Hakim et al.^28^, *Enterococcus*, with a relative abundance of more than 30%, predicted a significantly greater risk of subsequent febrile neutropenia and diarrheal illness in leukaemia patients.

We found a significant decrease in important bacteria responsible for producing metabolites, such as *Bacteorides*,* Faecalibacterium*,* Brevundimonas*, and *Pseudomonas*. These bacteria are key in producing metabolites and short-chain fatty acids (SCFAs) essential for maintaining intestinal balance. SCFAs like butyrate, propionate, and acetate are crucial metabolites produced in the colon, which provide energy for the intestinal lining. They help to strengthen the mucosal barrier and are known for their cancer-preventive and anti-inflammatory properties. A decrease in these beneficial bacteria could compromise the integrity of the gut barrier, potentially leading to intestinal damage and increasing the risk of bacterial translocation, as observed with *Enterococcus* and *Staphylococcus* entering the bloodstream. Consequently, the absence of these protective bacteria could compromise gut barrier integrity, making cancer patients more vulnerable to *Enterococcus* infections. Our linear discriminant analysis showed that cancer patients had higher *Enterococcus* levels and lower metabolite-producing bacteria such as *Prevotella*,* Dialister*,* Oscillospira*,* Megasphaera*,* Collinsella*, and *Faecalibacterium*. These changes are significant indicators of microbial imbalances in cancer patients. Importantly, the presence of the genus *Enterococcus* could potentially be used as a biomarker for disease in cancer patients, as consistently observed across various studies in colorectal cancer, lung cancer, and acute lymphoblastic leukaemia.

While our study has provided helpful information, it had its limitations. Firstly, we could not recruit a matched healthy control group due to COVID-19 restrictions, so we obtained the dataset for healthy individuals from published articles on Malaysian populations with the authors’ consent. Additionally, our study included cancer patients with various malignancies due to recruitment challenges associated with specific cancers, resulting in a small sample size for each cancer type. As a result, we could not fully explore the impact of different cancer types on gut microbiota composition. Although we confirmed no distinct clustering between cancer types and noted interindividual variability in our samples, we recognise that different cancer types may have varying influences on the gut microbiota.

Another limitation of this study was the inability to collect detailed clinical data, such as cancer stage, number of chemotherapy cycles, types of treatment protocols, and specific cancer types in some of our unclassified samples, due to ethical approval limitations, which restricted access to the patients’ medical records. Therefore, we could not fully evaluate patients’ general health and immunocompromised status at the time of sample collection. This information is crucial for understanding the cause and effect of gut microbiota dysbiosis in cancer patients. Future research should consider these factors based on our baseline findings when designing the methodology.

Additionally, we did not collect dietary information, a major factor influencing gut microbiota composition. While Malaysia is a multicultural country where various ethnic groups may share similar nutritional and cultural aspects, the potential impact of diet on our findings cannot be discounted. Dietary habits can vary significantly among cancer patients compared to the general population, and these variations could affect gut microbiota composition. Moreover, comparing dietary influences on gut microbiota between Malaysian cancer patients and those from West and East Asia could provide valuable insights into how cultural and nutritional differences impact gut microbiota in cancer patients. Moreover, employing longitudinal approaches will enable the capture of variations and changes in dietary patterns following symptoms and side effects of treatment. Another potential research avenue is to conduct an interventional study. Implementing controlled dietary interventions will allow us to observe the direct impact of specific diets on microbiome composition.

Acknowledging and addressing these limitations in future studies would enhance our understanding of the relationship between diet, gut microbiota, and cancer. Likewise, our study lacks a longitudinal approach, which would significantly improve our understanding of the impact of cancer and treatment on the gut microbiota of cancer patients. Conducting longitudinal studies would require patient follow-up and can be challenging due to the potential lack of cooperation. However, such studies are crucial as they allow access to the dynamic changes in the gut microbiota over time, not only in response to cancer treatment but also with the development of symptoms in cancer patients. Furthermore, since our participants were recruited from a single hospital, we could not assess potential regional variations in gut microbiota. Future studies should address this limitation by including a multi-centre sample collection to validate our findings. Collecting samples from multiple hospitals would provide a broader and more representative dataset, accounting for geographical and environmental differences that could influence gut microbiota composition. Additionally, applying machine learning (ML) approaches to identify microbial patterns predictive of metastases or response to treatment represents a promising avenue for future research. Machine learning techniques can analyse complex datasets to uncover hidden patterns and relationships that traditional methods might overlook. By leveraging these advanced analytical tools, researchers can potentially identify specific microbial signatures associated with disease progression and treatment outcomes.

## Conclusion

In conclusion, this study emphasises the significant differences in gut microbiota diversity and composition between cancer patients and healthy individuals. Our findings align with patients with inflammatory bowel disease (IBD), characterised by an expansion in the *Bacillota*/*Bacteroidotas* ratio^53^. While we generally do not observe significant differences in the microbial composition in patients receiving treatment and pre-treatment, diversity analysis confirmed microbial imbalances between cancer patients and healthy controls. These imbalances were particularly pronounced in symptomatic patients who exhibited gut dysbiosis. We also noted a notable increase in *Enterococcus* abundance in cancer patients, especially those with symptoms, with a reduction in beneficial bacteria. This overabundance of *Enterococcus*, a hallmark of microbial imbalance, has been documented in numerous studies and could be considered a potential biomarker for cancer patients. Future research is needed to elucidate the mechanisms through which *Enterococcus* influences cancer progression and treatment outcomes. Conducting longitudinal studies and addressing the limitations highlighted in this study will provide a deep understanding of the relationship between gut microbiota and cancer.

## Electronic supplementary material

Below is the link to the electronic supplementary material.

Supplementary Material 1

## Data Availability

The raw sequence data generated in this study have been deposited in the Genome Sequence Archive at the National Genomics Data Center, China National Center for Bioinformation/Beijing Institute of Genomics, Chinese Academy of Sciences. These data are publicly accessible at https://ngdc.cncb.ac.cn/gsa-human (GSA-Human: HRA006733). The raw sequence reads for the control group [20] were also used with the authors’ consent and permission.
