# Supplementary material for: Characterisation of gut microbiota in Malaysian cancer patients using V3-V4 region of 16S rRNA gene sequencing
Source: Sci Rep. 2025 Jul 1;15:21723. doi: 10.1038/s41598-025-06983-x (PMC12216863; doi:10.1038/s41598-025-06983-x)
Supplement: Supplementary file 1 — Supplementary Material 1 [file 41598_2025_6983_MOESM1_ESM.docx]

**Supplementary Information**

**Table S1** Total sequence frequency per sample retained for downstream analysis.

| **SAMPLE ID** | **Raw Reads** | **Filtered Reads** | **Reads Retained (%)** |
| --- | --- | --- | --- |
| RN-C10-S12 | 200583 | 168,864 | 84.19 |
| RN-A1-S17 | 189266 | 153,579 | 81.14 |
| RN-C12-S14 | 194748 | 140,648 | 72.22 |
| RN-A3-S19 | 196486 | 91,118 | 46.37 |
| RN-C1-S3 | 180360 | 86,854 | 48.16 |
| RN-A6-S22 | 156595 | 86,809 | 55.44 |
| RN-A4-S20 | 119268 | 85,855 | 71.98 |
| RN-C2-S4 | 160779 | 83,116 | 51.70 |
| RN-C4-S6 | 136657 | 83,039 | 60.76 |
| RN-A16-S32 | 130028 | 78,250 | 60.18 |
| RN-A11-S27 | 114599 | 73,429 | 64.07 |
| RN-C11-S13 | 124919 | 72,210 | 57.81 |
| RN-C13-S15 | 115303 | 70,726 | 61.34 |
| C013-0-S19 | 15845 | 65,711 | 414.71 |
| RN-C14-S16 | 100432 | 65,537 | 65.26 |
| RN-A14-S30 | 95286 | 60,979 | 64.00 |
| RN-SS021-S64 | 101904 | 59,372 | 58.26 |
| RN-A2-S18 | 85408 | 55,553 | 65.04 |
| RN-A8-S24 | 91725 | 53,494 | 58.32 |
| RN-A20-S88 | 68311 | 52,601 | 77.00 |
| C108-0-S27 | 87656 | 47,166 | 53.81 |
| RN-A5-S21 | 82400 | 47,047 | 57.10 |
| RN-A12-S28 | 76581 | 46,659 | 60.93 |
| RN-SS032-S69 | 67682 | 46,580 | 68.82 |
| RN-SS050-S77 | 75125 | 46,450 | 61.83 |
| RN-C5-S7 | 86601 | 46,161 | 53.30 |
| RN-SS025-S67 | 71062 | 43,176 | 60.76 |
| RN-SS047-S76 | 69237 | 42,167 | 60.90 |
| RN-SS053-S80 | 75577 | 41,322 | 54.68 |
| RN-A13-S29 | 70953 | 40,579 | 57.19 |
| RN-SS040-S2 | 61347 | 39,755 | 64.80 |
| RN-C9-S11 | 102229 | 39,269 | 38.41 |
| RN-C7-S9 | 65846 | 38,430 | 58.36 |
| RN-SS026-S68 | 66231 | 37,998 | 57.37 |
| RN-A10-S88 | 84220 | 37,899 | 45.00 |
| RN-A17-S88 | 60625 | 37,626 | 62.06 |
| C021-0-S21 | 66939 | 37,338 | 55.78 |
| RN-SS041-S73 | 62935 | 36,771 | 58.43 |
| RN-A15-S88 | 63926 | 36,548 | 57.17 |
| RN-SS030-S72 | 64806 | 35,777 | 55.21 |
| D025-0-S29 | 65086 | 35,622 | 54.73 |
| RN-A9-S25 | 52471 | 35,594 | 67.84 |
| RN-SS033-S70 | 60140 | 35,037 | 58.26 |
| C043-0-S24 | 53679 | 34,863 | 64.95 |
| RN-SS024-S66 | 57363 | 34,705 | 60.50 |
| RN-SS042-S74 | 58152 | 34,236 | 58.87 |
| C056-0-S26 | 55470 | 33,341 | 60.11 |
| B090-0-S16 | 50642 | 32,509 | 64.19 |
| RN-C8-S10 | 80889 | 32,445 | 40.11 |
| RN-SS020-S1 | 69639 | 30,904 | 44.38 |
| RN-SS054-S81 | 54486 | 30,556 | 56.08 |
| RN-SS051-S78 | 60620 | 30,350 | 50.07 |
| RN-SS022-S65 | 60473 | 29,970 | 49.56 |
| RN-SS045-S75 | 58999 | 29,530 | 50.05 |
| RN-C3-S5 | 67000 | 27,153 | 40.53 |
| C026-0-S22 | 51343 | 26,154 | 50.94 |
| RN-SS018-S62 | 57057 | 25,899 | 45.39 |
| RN-SS052-S3 | 54001 | 23,294 | 43.14 |
| RN-A19-S34 | 54344 | 22,998 | 42.32 |
| RN-C6-S8 | 52561 | 18,579 | 35.35 |
| A077-0-S9 | 40189 | 18,325 | 45.60 |
| D163-0-S43 | 30650 | 18,272 | 59.62 |
| B032-0-S13 | 35100 | 17,392 | 49.55 |
| RN-A7-S88 | 44021 | 16,991 | 38.60 |
| A013-0-S7 | 32750 | 14,302 | 43.67 |
| D099-0-S38 | 24739 | 11,204 | 45.29 |
| B069-0-S14 | 15839 | 9,583 | 60.50 |
| D057-0-S33 | 19159 | 8,857 | 46.23 |
| B021-0-S12 | 10753 | 6,074 | 56.49 |
| D077-0-S37 | 8033 | 4,273 | 53.19 |
